# Co-expression of Cyanobacterial Genes for Arsenic Methylation and Demethylation in *Escherichia coli* Offers Insights into Arsenic Resistance

**DOI:** 10.3389/fmicb.2017.00060

**Published:** 2017-01-24

**Authors:** Yu Yan, Xi-Mei Xue, Yu-Qing Guo, Yong-Guan Zhu, Jun Ye

**Affiliations:** ^1^Key Lab of Urban Environment and Health, Institute of Urban Environment – Chinese Academy of SciencesXiamen, China; ^2^University of Chinese Academy of SciencesBeijing, China; ^3^Fujian Provincial Key Laboratory of Agroecological Processing and Safety Monitoring, College of Life Sciences, Fujian Agriculture and Forestry UniversityFuzhou, China; ^4^State Key Lab of Urban and Regional Ecology, Research Center for Eco-environmental Sciences – Chinese Academy of SciencesBeijing, China

**Keywords:** arsenic methylation, arsenic demethylation, arsenic resistance, MAs(III) antibiotic, *Nostoc* sp. PCC 7120

## Abstract

Arsenite [As(III)] and methylarsenite [MAs(III)] are the most toxic inorganic and methylated arsenicals, respectively. As(III) and MAs(III) can be interconverted in the unicellular cyanobacterium *Nostoc* sp. PCC 7120 (*Nostoc*), which has both the *arsM* gene (*NsarsM*), which is responsible for arsenic methylation, and the *arsI* gene (*NsarsI*), which is responsible for MAs(III) demethylation. It is not clear how the cells prevent a futile cycle of methylation and demethylation. To investigate the relationship between arsenic methylation and demethylation, we constructed strains of *Escherichia coli* AW3110 (Δ*arsRBC*) expressing *NsarsM* or/and *NsarsI*. Expression of *NsarsI* conferred MAs(III) resistance through MAs(III) demethylation. Compared to NsArsI, NsArsM conferred higher resistance to As(III) and lower resistance to MAs(III) by methylating both As(III) and MAs(III). The major species found in solution was dimethylarsenate [DMAs(V)]. Co-expression of *NsarsM* and *NsarsI* conferred As(III) resistance at levels similar to that with *NsarsM* alone, although the main species found in solution after As(III) biotransformation was methylarsenate [MAs(V)] rather than DMAs(V). Co-expression of *NsarsM* and *NsarsI* conferred a higher level of resistance to MAs(III) than found with expression of *NsarsM* alone but lower than expression of only *NsarsI.* Cells co-expressing both genes converted MAs(III) to a mixture of As(III) and DMAs(V). In *Nostoc NsarsM* is constitutively expressed, while *NsarsI* is inducible by either As(III) or MAs(III). Thus, our results suggest that at low concentrations of arsenic, NsArsM activity predominates, while NsArsI activity predominates at high concentrations. We propose that coexistence of *arsM* and *arsI* genes in *Nostoc* could be advantageous for several reasons. First, it confers a broader spectrum of resistance to both As(III) and MAs(III). Second, at low concentrations of arsenic, the MAs(III) produced by NsArsM will possibly have antibiotic-like properties and give the organism a competitive advantage. Finally, these results shed light on the role of cyanobacteria in the arsenic biogeochemical cycle.

## Introduction

Arsenic is a ubiquitous environmental toxin, and primarily occurs in inorganic forms, including arsenate [As(V)] and arsenite [As(III)] (Zhu et al., 2014). Because of the unavoidable exposure to arsenic, nearly all of the living organisms have arsenic detoxifying systems (Rosen, 2002; Liu et al., 2013). As(III) eﬄux and oxidation are considered to be efficient detoxification pathways in Bacteria and Archaea (Páez-Espino et al., 2009). As(III) methylation catalyzed by As(III) *S*-adenosylmethionine methyltransferase (ArsM) has also been shown to confer resistance to As(III) by the eventual production of less toxic pentavalent methylated species, including methylarsonate [MAs(V)], dimethylarsonate [DMAs(V)], trimethylarsine oxide [TMAsO], or volatile methylated arsenicals (Levy et al., 2005; Challenger, 2006; Qin et al., 2006). However, the trivalent methylated arsenic species, methylarsenite [MAs(III)] and dimethylarsenite [DMA(III)], which are the immediate products of As(III) biomethylation (Dheeman et al., 2014; Marapakala et al., 2015), are far more toxic than As(III) (Petrick et al., 2000; Mass et al., 2001). MAs(V) can also be reduced to MAs(III) in some bacteria (Yoshinaga et al., 2011), so other microbes have evolved various processes to detoxify MAs(III) as it is formed. MAs(III), like As(III), is detoxified by oxidation or eﬄux in some microbes. A NADPH-FMN dependent oxidoreductase (ArsH) and a membrane permease (ArsP) are responsible for MAs(III) oxidation and eﬄux, respectively (Chen et al., 2015a,b). Furthermore, MAs(III) demethylation catalyzed by a C-As lyase (ArsI) is an effective MAs(III) detoxification pathway (Yoshinaga and Rosen, 2014).

Demethylation reverses methylation by converting methylated arsenic into inorganic arsenic. Since both ArsM and ArsI can use MAs(III) as substrate, they may compete with each other for MAs(III). Intuitively, it would seem that, if both methylation and demethylation occur in the same organism, a futile cycle could be created. Nevertheless, the genes for arsenic methylation and demethylation coexist in some microorganisms. For example, *Nostoc* can both methylate As(III) and demethylate MAs(III) and has both the *NsarsM* and *NsarsI* genes in its chromosome (Yin et al., 2011; Yan et al., 2015). However, it is not known whether coexistence of *arsM* and *arsI* creates a futile cycle of arsenic methylation and demethylation or whether it enhances resistances to selected arsenicals.

In cyanobacteria, morphological, physiological and biochemical changes, genomics, and proteomics have been found to be involved in response to arsenic stress (Srivastava et al., 2009; Pandey et al., 2012; Sánchez-Riego et al., 2014). Furthermore, various cyanobacteria were shown to be able to accumulate and resist arsenic by multiple arsenic biotransformations (Yin et al., 2012; Wang et al., 2013). For instance, in *Synechocysis* sp. PCC 6803, which is the best-understood cyanobacterium on arsenic resistance and metabolism (Huertas et al., 2014), an *ars* operon (*arsBHC*) mediates the main arsenic resistance mechanism (López-Maury et al., 2003), and another two arsenate reductases (*arsI1* and *arsI2*) (López-Maury et al., 2009) and an arsenite methyltransferase (*arsM*) (Yin et al., 2011) were also found to be the resistance determinants. Similarly, *Nostoc* is also a complex system for analysis of arsenic biotransformations. In addition to methylation and demethylation, *Nostoc* has other arsenic biotransformation pathways, in particular As(V) reduction (Pandey et al., 2013), As(III) oxidation, and eﬄux systems. Thus, to investigate the relationship between As(III) methylation and MAs(III) demethylation, we constructed a simplified model using arsenic-hypersensitive *Escherichia coli* AW3110 strains (Δ*arsRBC*) expressing *NsarsM* or/and *NsarsI*. The effect of co-expression on biotransformations of and resistance to As(III) and MAs(III) was examined. The kinetic parameters of NsArsM for As(III) and MAs(III) and regulations of *NsarsM* and *NsarsI* in *Nostoc* were also evaluated to provide a more comprehensive understanding of the arsenic methylation cycle.

## Materials and Methods

### Bacterial Strains, Media, and Chemical Reagents

*Escherichia coli* strains were aerobically cultivated in Lysogeny Broth (LB) medium (Sambrook et al., 1989), supplemented with required antibiotics at 37°C with shaking at 180 rpm. *E. coli* strain DH5α (Promega, Madison, WI, USA) was used for plasmid construction and replication, and Rosetta (DE3) (Novagen, Madison, WI, USA) was used for protein expression. *E. coli* strain AW3110 (DE3) [Δ*arsRBC*; ArsR-repressor, ArsB-As(III) eﬄux pump, ArsC-As(V) reductase] was used for arsenic resistance and biotransformation assays (Carlin et al., 1995). *Nostoc* (also known as *Anabaena*), kindly provided by Professor Wen-Li Chen, Huazhong Agricultural University, was grown in BG11 medium without nitrate and cultured as previously described (Yan et al., 2015). MAs(III) was produced by reduction of MAs(V) using Na_2_S_2_O_3_, Na_2_S_2_O_5_, and H_2_SO_4_ (Yoshinaga and Rosen, 2014). All other used reagents were purchased from commercial sources, and were of analytical grade or better.

### Construction of *E. coli* AW3110 Strain Containing *NsarsM* and *NsarsI*

*NsarsM* (*alr3095*, accession number HQ891148) and *NsarsI* (*alr1104*, accession number BAB73061) have been identified in previous studies (Yin et al., 2011; Yan et al., 2015). The *NsarsM* or *NsarsI* was cloned into the expression vector pET28a (Novagen, Madison, WI, USA) or pET22b (Novagen, Madison, WI, USA) to generate the plasmid pET28a-*NsarsM*, pET28a-*NsarsI*, or pET22b-*NsarsI*; and the primers used for amplification were listed in **Table 1**. The two pET28a plasmids were transformed independently into *E. coli* AW3110 to construct strains expressing *NsarsM* or *NsarsI*. The plasmids pET28a-*NsarsM* and pET22b-*NsarsI* were co-transformed into *E. coli* AW3110. Although the two-plasmid system using the same origin of replication are commonly believed not to exist in one *E. coli* cell, several similar approaches with two incompatible plasmids were successfully used if under the selection pressure of two different antibiotics (Yang et al., 2001; Wang et al., 2007; Zhang et al., 2011). Thus, the strain co-expressing *NsarsM* and *NsarsI* was selected by growth on LB agar plate containing 100 μg mL^-1^ ampicillin, 50 μg mL^-1^ kanamycin, and 30 μg mL^-1^ chloramphenicol (pET28a-*NsarsM* and pET22b-*NsarsI* plasmids confer kanamycin and ampicillin resistances, respectively).

**Table 1:** Primers used in this study.

Primer	Sequence (5′–3′)	Feature
*NsarsM*-pET28a-F	CCATGGCAACCTATTTAGAAACAGC (*Nco*I site underlined)	pET28a-*arsM* plasmid construction
*NsarsM*-pET28a-R	CTCGAGACAGCAACCACCACCGTTATAATG (*Xho*I site underlined)	
*NsarsI*-pET28a-F	CCATGGCATCCGTTATGAAAACACACG (*Nco*I site underlined)	pET28a-*arsI* and pET22b-*arsI* (Yan et al., 2015) plasmids construction
*NsarsI*-pET22b-F	CATATGTCCGTTATGAAAACACACG (*Nde*I site underlined)	
*NsarsI*-pET-R	CTCGAGAGCACAACATGACTTC (*Xho*I site underlined)	
*NsarsM*-qpcr-F	TTTACCTGTGGCTGATGG	RT-qPCR: *NsarsM* (LOCUS: HQ891147) transcript
*NsarsM*-qpcr-R	TTCTGGCATAGGCACTTT	
*NsarsI*-qpcr-F	AAACCGACTACGCTAAAT	RT-qPCR: *NsarsI* (LOCUS: BA000019) transcript
*NsarsI*-qpcr-R	CTTCTTGACAGCCTGAAT	
*NsrnpB*-qpcr-F	AGGGAGAGAGTAGGCGTTGG	RT-qPCR: *NsrnpB* (LOCUS: X65648) transcript (Latifi et al., 2005)
*NsrnpB*-qpcr-R	GGTTTACCGAGCCAGTACCTCT	

### Western Blot Analysis

Western blots were used to detect expression of *NsarsM* or/and *NsarsI* in the *E. coli* AW3110 strains. *E. coli* AW3110 cells in exponential phase were induced by 1 mM isopropyl β-d-1-thiogalactopyranoside (IPTG). The proteins from cell lysate were separated by sodium dodecyl sulfate-polyacrylamide gel electrophoresis (SDS-PAGE; gradient 15%), and transferred to polyvinylidene fluoride (PVDF) membranes (Pall Corporation, East Hills, NY, USA) using the Trans-Blot Turbo Transfer System (Bio-Rad, Hercules, CA, USA). Immunoblot analyses were carried out with anti-His tag (D3I1O, Cell Signaling, Beverly, MA, USA) by incubating at 4°C overnight. Membranes were washed, and incubated with appropriate peroxidase-conjugated secondary antibody. Specific bands were visualized by WesternBright ECL HRP substrate (Advansta, Inc., Menlo Park, CA, USA), and finally scanned on a Kodak image station 4000 mm Pro (Carestream Health, New Haven, CT, USA).

### Arsenic Resistance Assays

As(III) and MAs(III) resistance assays of *E. coli* AW3110 strains bearing pET28a vector, pET28a-*NsarsM*, pET28a-*NsarsI*, or pET28a-*NsarsM+*pET22b-*NsarsI* plasmids were performed as described previously (Ye et al., 2014; Yan et al., 2015). The cells were cultured in LB medium containing As(III) (0, 10, 30, 50, 70, 90, and 110 μM) at 37°C or in ST medium (10-fold concentrated ST 10^-1^ medium) (Maki et al., 2006) containing MAs(III) (0, 1, 2, 3, 4, 6, and 8 μM) at 30°C. After incubating for 24 h, optical density at 600 nm (OD_600__nm_) was measured using an ultraviolet-visible spectrophotometer (UV-6300 double beam spectrophotometer, Mapada, Shanghai, China). In addition, the four *E. coli* AW3110 strains were treated with 35 μM As(III) and 2 μM MAs(III) at the same time in ST medium, and OD_600__nm_ was monitored at 0, 3, 6, 12, 18, 24, 30 h.

### Arsenic Biotransformation in *E. coli* AW3110 Strains

The IPTG-induced *E. coli* AW3110 cells were cultured in LB medium containing 25 μM As(III) at 37°C for 24 h or ST 10^-1^ medium containing 1 μM MAs(III) or 1 μM As(III) and 0.5 μM MAs(III) at 25°C for 1 h. Same amounts of arsenicals were also added to LB medium or ST 10^-1^ medium without cells as non-inoculated controls. All samples were centrifuged at 13400 *g* for 2 min, and the supernatants were collected. Arsenic speciation was determined by high-performance liquid chromatography (HPLC, 1200, Agilent Technologies, Palo Alto, CA, USA)-inductively coupled plasma-mass spectrometry (ICP-MS, 7500a, Agilent Technologies, Palo Alto, CA,USA) to analyze arsenic biotransformation.

### Purification of NsArsM

The plasmid pET28a-*NsarsM* was transformed into *E. coli* strain Rosetta (DE3) for purification of NsArsM. His-tagged NsArsM expression was induced by addition of 1 mM IPTG when OD_600__nm_ of *E. coli* Rosetta (DE3) cells reached 0.5–0.8. The induced cells were ruptured in a French-press at 10 MPa. NsArsM was purified by Ni-NTA agarose column (Qiagen, Hilden, Germany) according to the manufacturer’s instruction. The purified NsArsM was concentrated by 10-kDa cutoff Amicon Ultrafilter (Millipore, Bedford, MA, USA), and identified using SDS-PAGE.

### Enzyme Kinetics of NsArsM

In order to determine kinetic parameters, *in vitro* As(III) and MAs(III) reaction systems of NsArsM were established according to previous study with a few modifications (Qin et al., 2006). For the methylation reaction, 8 μM purified NsArsM was added in a buffer consisting of 50 mM MOPS buffer (pH 7.5) with 125 mM NaCl, 1 mM *S*-adenosylmethionine chloride (SAM), 8 mM reduced glutathione (GSH), and the indicated concentrations of As(III) or MAs(III). After the reaction was performed at 37°C for 1 h, the assay was immediately terminated by boiling, and added 3% H_2_O_2_ to oxidize arsenic. Enzyme activity was measured by determining arsenic species. The mole equivalents of methyl groups (-CH_3_) transferred from SAM to arsenic were used to approximate the apparent rates of methylation. Since ArsM catalyzes three separate methylation reactions, the overall rates were lumped together as one mole of SAM methyl groups to methylate one mole of As(III) to MAs, two moles of SAM to methylate As(III) to DMAs, and three moles of SAM to methylate to TMAs (Walton et al., 2003; Wang et al., 2012). Non-linear regression analysis was performed with OriginPro 8.5.

### Arsenic Speciation Analysis

Samples were filtered through 0.22 μm filters (Millipore, Bedford, MA, USA), and analyzed by HPLC-ICP-MS using previously established instrument parameters (Zhu et al., 2008). Arsenic species were determined with either a 10-μm PRP-X100 anion exchange column (250 mm × 4.1 mm ID, Hamilton, Reno, NV, USA) eluted isocratically with a mobile phase (pH 6.2) consisting of 10 mM ammonium di-hydrogen phosphate and 10 mM ammonium nitrate (Ye et al., 2014) or a Jupiter 5 μ C18 300A reverse-phase column (250 mm × 4.6 mm, Phenomenex, Torrance, CA, USA) using the mobile phase (pH 5.95) with 3 mM malonic acid, 5 mM tetrabutylammonium hydroxide, and 5% methanol (Yoshinaga and Rosen, 2014). The flow rate for HPLC was 1.0 mL min^-1^, and ICP-MS was tuned for monitoring of m/z 75 (arsenic). Arsenic species in samples were identified by retention times which were compared with those of the standards. The arsenic was quantified by external calibration curves with peak areas integrated by using WinFASS.

### RNA Isolation and Reverse Transcription-Quantitative Real-Time PCR (RT-qPCR)

*Nostoc* at the mid-exponential growth phase was cultured with or without arsenic for 6 h. Total RNA was extracted from *Nostoc* cells treated with As(III) (0, 1, 5, 10, 40, and 100 μM) or MAs(III) (0, 0.2, 1, 3, 6, and 12 μM) by using TRIzol reagent (Invitrogen Life Technologies, Gaithersburg, MD, USA) following the manufacturer’s recommendations. Contaminating genomic DNA was removed from total RNA using DNase I (Promega, Madison, WI, USA) at 37°C for 45 min. The total RNA was further purified with RNA clean kit (Tiangen, Beijing, China). About 40 ng purified RNA was used for RT-qPCR with GoTaq^®^ 1-Step RT-qPCR System (Promega, Madison, WI, USA) in a 20-μL volume. The primers for *NsarsM* and *NsarsI* were listed in **Table 1**. qPCR was performed on a LightCycler 480 (Roche Applied Science, Indianapolis, IN, USA). Each reaction was carried out in triplicate with the housekeeping gene *rnpB* as the internal standard (Vioque, 1992; Latifi et al., 2005). The PCR efficiencies of the targets (*NsarsM* and *NsarsI*) and reference (*rnpB*) were calculated from the slope of their standard curves (*E* = 10^[-1/slope]^), respectively, and the relative transcript levels of *NsarsM* and *NsarsI* were calculated using the formula (E_target_)^ΔCp^target^(control-sample)^ × (E_ref_)^ΔCp^ref^(sample-control)^ (Pfaﬄ, 2001).

## Results

### Gene Expression in *E. coli* AW3110 Strains

**FIGURE 1:**
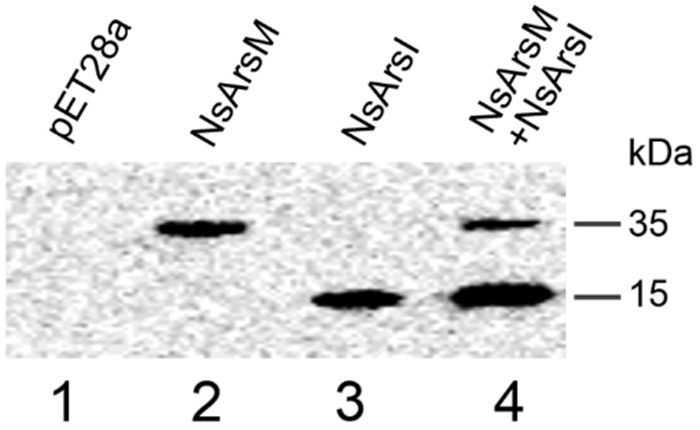
**Western-blot analysis of NsArsM and NsArsI.** lane1 through 4 contained proteins extracted from *Escherichia coli.* AW3110 cells bearing the empty vector pET28a (lane1) and the following vectors containg the *NsarsM* and/or *NsarsI* genes: pET28a-*NsarsM* (lane2); pET28a-*NsarsI* (lane3); and pET28a-*NsarsM* + pET22b-*NsarsI* (lane4). The blots were probed with anti-His tag, followed by peroxidase-conjugated secondary antibody.

To investigate arsenic resistance and biotransformation when NsArsM and NsArsI co-existed in the same cell, *E. coli* AW3110 strain co-expressing *NsarsM* and *NsarsI* genes was constructed. The *E. coli* AW3110 strain bearing plasmid pET28a served as negative control. The expression levels of *NsarsM* and *NsarsI* in the single or co-expressed *E. coli* AW3110 cells were estimated by western blot using anti-His antibodies that recognize NsArsM or NsArsI (**Figure 1**). The results show that both *NsarsM* and *NsarsI* were expressed, separately and together. When co-expressed, more NsArsI was produced than NsArsM.

### Arsenic Resistance Assays

**FIGURE 2:**
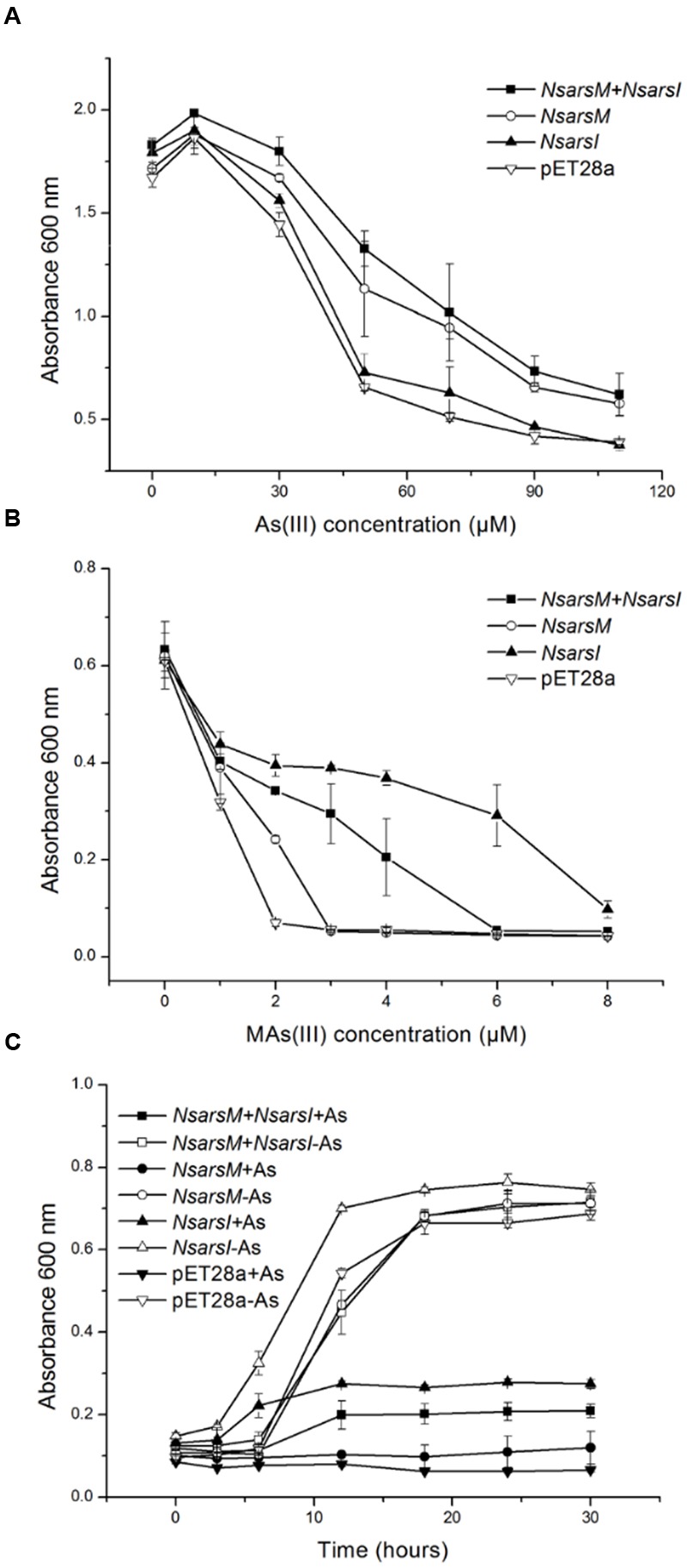
**Arsenic resistance of *E. coli* AW3110 bearing pET28a, pET28a-*NsarsM*, pET28a-*NsarsI* and pET28a-*NsarsM*+ pET22b-*NsarsI* plasmids.** The four *E. coli* AW3110 strains were incubated with the indicated concentrations of As(III) **(A)** or MAs(III) **(B)**. Cell growth was monitored by spectrophotometry at 600 nm. Filled squares, plasmids pET28a-*NsarsM*+ pET22b-*NsarsI*; open circles, plasmid pET28a-*NsarsM*; filled triangles, plasmid pET28a-*NsarsI*; open inverted triangles, plasmid pET28a. **(C)** Sensitivity of the four *E. coli* AW3110 strains to the mixture of As(III) and MAs(III). The growth curves are shown for LB media containing the mixture of 35 μM As(III) and 2 μM MAs(III) (+As) or without arsenic (-As). Squares, plasmids pET28a-*NsarsM*+ pET22b-*NsarsI*; circles, plasmid pET28a-*NsarsM*; triangles, plasmid pET28a-*NsarsI*; inverted triangles, plasmid pET28a. Filled symbols, 35 μM As(III) + 2 μM MAs(III); open symbols, 0 μM As(III) + 0 μM MAs(III). The error bars represent the standard error from three independent biological experiments.

Resistance assays were conducted using *E. coli* AW3110 expressing *NsarsM* and/or *NsarsI*. When *E. coli* AW3110 cells were cultured in arsenic-free medium, the growth rates of all four *E. coli* AW3110 strains were nearly the same (**Figure 2**). In As(III) resistance assays, the growth of all the *E. coli* AW3110 cells was stimulated by 10 μM As(III), but inhibited when the As(III) concentration was over 30 μM (**Figure 2A**). The reason for this apparent growth stimulation by As(III) is not known, but it has been observed in another alga (Zhang et al., 2013). Furthermore, both the *E. coli* AW3110 cells expressing *NsarsM* and cells co-expressing *NsarsM* and *NsarsI* grew much better than those bearing pET28a or pET28a*-NsarsI* at 50–110 μM of As(III) (*t*-test, *P* < 0.05). For MAs(III) resistance assays, 1 μM MAs(III) inhibited growth of all of *E. coli* AW3110 cells (**Figure 2B**). *E. coli* AW3110 expressing *NsarsI* grew best at concentrations of MAs(III) between 2 and 8 μM, while *E. coli* AW3110 expressing *NsarsM* only grew better than the vector alone at 2 μM MAs(III). *E. coli* AW3110 co-expressing *NsarsM* and *NsarsI* exhibited less resistance to MAs(III) than that expressing *NsarsI*, and significantly higher resistance than that expressing *NsarsM*. In addition, the growth rates of *E. coli* AW3110 exposed to mixed 35 μM As(III) and 2 μM MAs(III) monitored over 30 h (**Figure 2C**), showed that co-expression of *NsarsM* and *NsarsI* conferred higher arsenic resistance than expression of *NsarsM*, but lower resistance than that of *NsarsI*.

### Arsenic Biotransformation in *E. coli* AW3110

**FIGURE 3:**
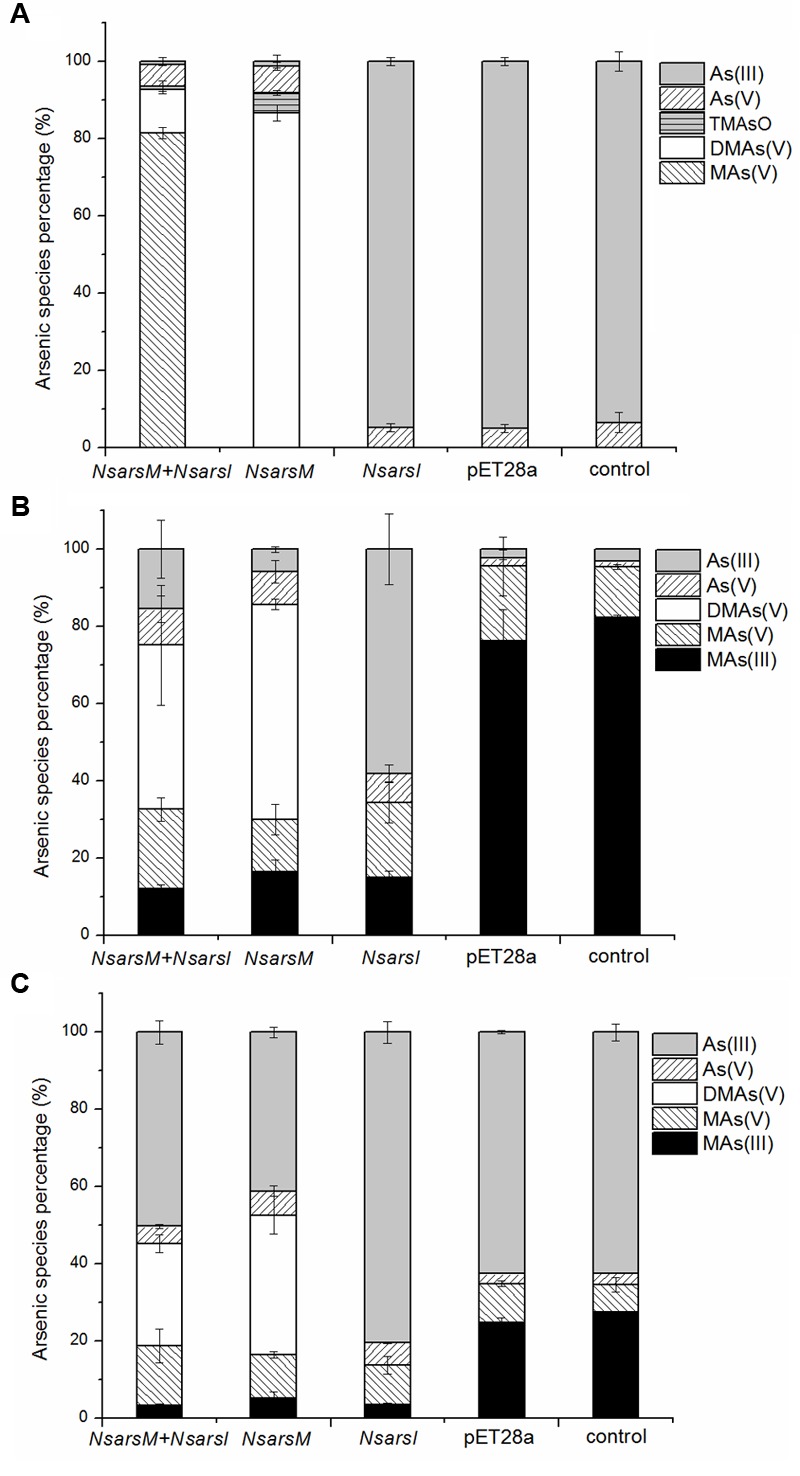
**The percentage of arsenic species [As(III), gray bars; As(V), left-hatched bars; TMAsO, horizontally hatched bars, DMAs(V), white bars; MAs(V), right-hatched bars; MAs(III), black bars] in media incubated *E. coli* AW3110 bearing pET28a-*NsarsM* + pET28a-*NsarsI* plasmids (*NsarsM*+*NsarsI*), pET28a-*NsarsM* (*NsarsM*), pET28a-*NsarsI* (*NsarsM*) and pET28a vector (pET28a) and non-incubated media (control) exposed to 25 μM As(III) for 24 h (A)**, 1 μM MAs(III) for 1 h **(B)**, or mixture of 1 μM As(III) and 0.5 μM MAs(III) for 1 h **(C)**. The error bars represent the standard error from three independent biological experiments.

To elucidate the biotransformation pathways when *NsarsM* or/and *NsarsI* were expressed in *E. coli* AW3110, arsenic species in As(III) and/or MAs(III)-containing media with or without cells were determined (**Figure 3**). When treated with 25 μM As(III), *E. coli* AW3110 bearing pET28a or pET28a-*NsarsI* did not change arsenic species in the media compared to the non-inoculated control. The detection of As(V) in the control may come from the trace contaminant of reagent and/or oxidation by air. While the cells expressing *NsarsM* or co-expressing *NsarsM* and *NsarsI* transformed As(III) into methylated arsenic (**Figure 3A**). It is worthwhile to note that the methylated species produced were different for the two strains. DMAs(V) and TMAsO were the predominant arsenic species, and MAs(V) was undetectable in the medium of cells expressing *NsarsM*. However, MAs(V) was the main arsenic species in the medium culturing *E. coli* AW3110 co-expressing *NsarsM* and *NsarsI*. When treated with 1 μM MAs(III) (**Figure 3B**), MAs(V) was detected in the controls, and it was probably coming from the spontaneous chemical oxidation of MAs(III) under aerobic conditions. DMAs(V) and As(III) were the primary arsenic species in the culture media of *E. coli* AW3110 cells expressing *NsarsI* and *NsarsM*, respectively. Both As(III) and DMAs(V) were detected in the medium with co-expression AW3110 strain. Similarly, under the exposure to a mixture of As(III) and MAs(III) (**Figure 3C**), DMAs(V) was only detected in the medium when *NsarsM* was expressed alone or co-expressed with *NsarsI*, and the percentage of As(III) increased in the latter.

### Reaction Kinetics of NsArsM

**FIGURE 4:**
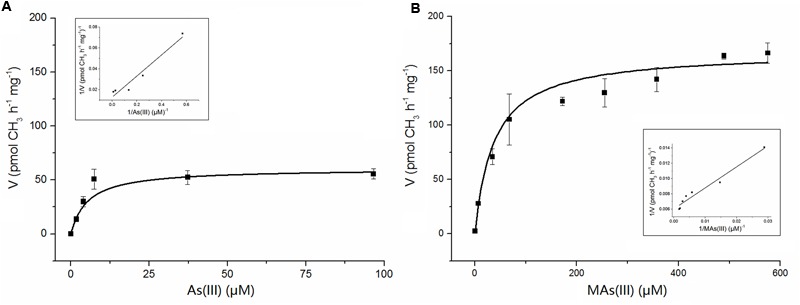
**The kinetic curves of NsArsM for As(III) (A)** and MAs(III) **(B)** methylation with 1 mM SAM as methyl donor, 8 mM GSH as reductant. Reaction velocity (*v*) is expressed as pmol CH_3_ transferred from SAM to arsenic per hour per mg of purified NsArsM. The lines show the least squares fit of Michaelis–Menten equation to the data. The error bars represent the standard error from three independent biological experiments. The insert figure showed the double reciprocal plots of the relation between the concentration of arsenic and the velocity.

Apparent kinetic constants were determined with purified NsArsM from the rate of methyl transfer from SAM. The relationship between the substrate As(III) or MAs(III) and the enzyme NsArsM fit conventional Michaelis–Menten kinetics (**Figure 4**). The affinity for As(III) was sevenfold greater than MAs(III), with a *K*_m_ of 5 ± 1 μM for As(III) and 37 ± 4 μM for MAs(III). The *V*_max_ for As(III) and MAs(III) were 60 ± 5 pmol CH_3_ h^-1^ mg^-1^ and 167 ± 6 pmol CH_3_ h^-1^ mg^-1^, respectively.

### Transcription of *NsarsM* and *NsarsI* in *Nostoc*

**FIGURE 5:**
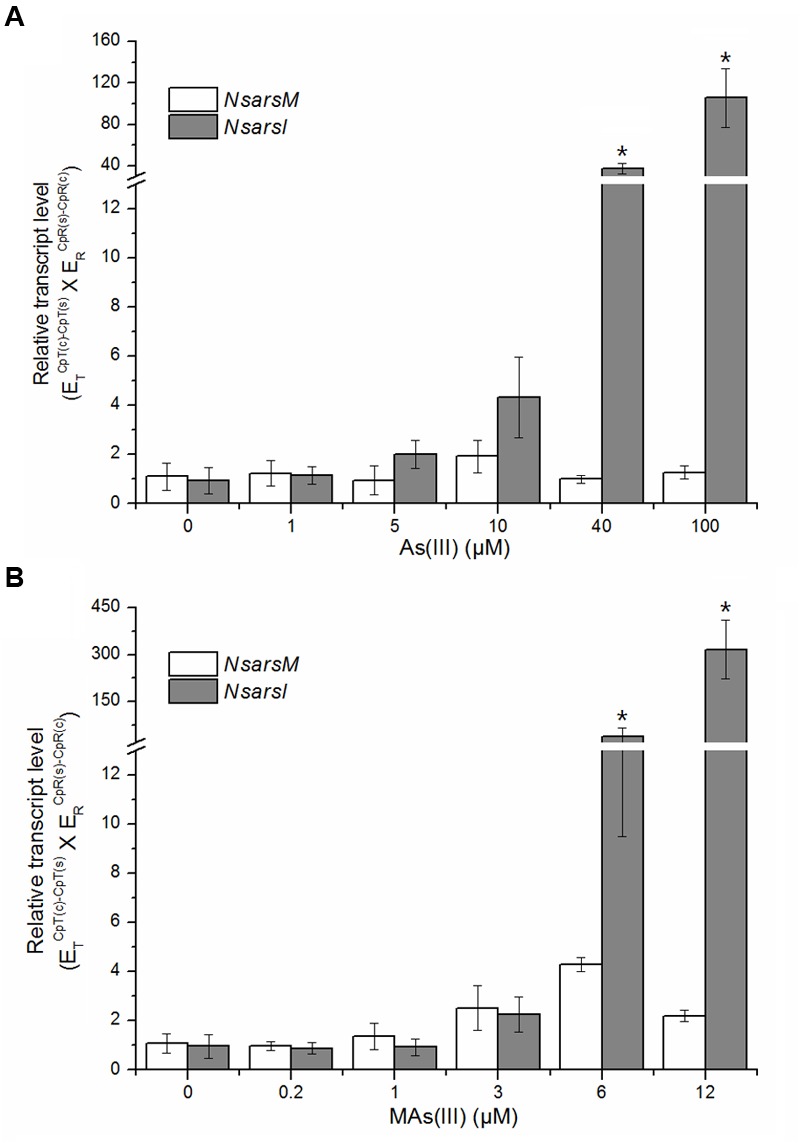
**Effects of arsenic on the transcript levels of *NsarsM* (white bars) and *NsarsI* (gray bars).** Mid-exponential *Nostoc* were exposed to indicated concentrations of As(III) **(A)** or MAs(III) **(B)** for 6 h, and *NsarsM* and *NsarsI* transcriptions were investigated by RT-qPCR analysis. The error bars indicate the standard error from three independent biological experiments. Asterisks represent significant difference (^∗^*P* < 0.05).

The transcript levels of *NsarsM* and *NsarsI* were analyzed by RT-qPCR after *Nostoc* exposed to MAs(III) or As(III) at indicated concentrations for 6 h. *Nostoc* under identical cultivation conditions without arsenic was used as a control. As shown in **Figure 5**, the transcript levels of *NsarsM* had no significant difference (*P* > 0.05) between the *Nostoc* cultures with and without arsenic. The transcript levels of *NsarsI* were not significantly increased (*P* > 0.05) by As(III) less than 40 μM or MAs(III) less than 6 μM, while they were significantly enhanced (*P* < 0.05) when 40 or 100 μM As(III), or 6 or 12 μM MAs(III) was added in the cultures.

## Discussion

Arsenic demethylation lowered the efficiency of arsenic methylation in the co-expressing *E. coli*. The arsenic methylation rate is an important factor to determine whether As(III) methylation is a detoxification mechanism (Rahman and Hassler, 2014). Cells of *E. coli* over-expressing various *arsM* genes rapidly methylate As(III) into DMAs(V) or TMAsO, conferring As(III) tolerance (Qin et al., 2006, 2009; Yin et al., 2011). Our results also showed that resistance of *E. coli* AW3110 expressing *NsarsM* to As(III) was significantly enhanced by methylating As(III) to DMAs(V) and TMAsO. However, when NsArsM and NsArsI coexisted, As(III) was mainly methylated to MAs(V) rather than DMAs(V). The result suggests that part of MAs dissociates from NsArsM as a methylated trivalent species. In *E. coli* AW3110 expressing *NsarsM*, free MAs(III) dissociated from NsArsM could bind to the enzyme again, and was further methylated to DMAs. While NsArsI coexisted with NsArsM, both of them may competitively bind to free MAs(III), MAs(III) released from NsArsM was more likely to be further oxidized to MAs(V). Herein, in addition to the direct transformation of methylated arsenic into inorganic species, competitive binding of ArsM and ArsI to MAs(III) may be another reason that arsenic demethylation limits methylation efficiency.

MAs(III) demethylation was slowed by arsenic methylation when *NsarsM* and *NsarsI* were co-expressed in *E. coli*. The competition between NsArsM and NsArsI with MAs(III) in co-expressed *E. coli* AW3110 may lead to the decrease of demethylation efficiency. The *K*_0.5_ value for MAs(III) of NsArsI was 7.55 μM in our previous study (Yan et al., 2015), and the *K*_m_ value of NsArsM for MAs(III) was 37 μM in this study. It indicated that MAs(III) has a higher affinity for NsArsI than NsArsM, and MAs(III) preferred to be demethylated rather than be methylated. Therefore, both DMAs(V) and As(III) were detected when *E. coli* AW3110 co-expressing *NsarsM* and *NsarsI* was treated with MAs(III). Even though MAs(III) demethylation was limited, co-expression of *NsarsM* and *NsarsI* exhibited higher MAs(III) resistance than single expression of *NsarsM* due to the high detoxification efficiency of NsArsI for MAs(III).

So far, we demonstrated that arsenic methylation and demethylation may limit each other in *E. coli* co-expressing *NsarsM* and *NsarsI*, and we hypothesized that the regulations of *NsarsM* and *NsarsI* may be different to avoid functioning simultaneously, thus entering a futile cycle of methylation and demethylation, in *Nostoc*. So we analyzed the genome sequences of *Nostoc*, and found that *NsarsM* is not adjacent to an *arsR*, while *NsarsI* is in an *ars* operon containing four genes: *asr1102* (homolog of arsenite eﬄux protein; *arsB*), *all1103* (transcriptional regulator; *arsR*), *alr1104* (*arsI*), and *alr1105* (arsenate reductase; *arsC*) (Pandey et al., 2012). ArsR that regulates the expression of the *ars* operon is responsive to both As(III) and MAs(III) (Chen et al., 2014). In coincidence with this, the RT-qPCR results showed that *NsarsI* was induced by higher arsenic concentrations [As(III) at 40 and 100 μM; MAs(III) at 6 and 12 μM], while the expression of *NsarsM* is constitutive at all arsenic concentrations. Furthermore, the main product was As(V) in *Nostoc* treated with As(III) up to 100 μM (Yin et al., 2011), suggesting that arsenic methylation may not be the primary pathway of As(III) detoxification. This was also supported by proteomic data which implied *arsM* seems not to be up-regulated when *Nostoc* treated with 40 μM As(V) (Pandey et al., 2012). Recently, MAs(III) as the initial product of ArsM was proposed to be a primitive antibiotic produced by organisms at the early Earth (Chen et al., 2015b; Li et al., 2016). Thus, we propose that NsArsM may predominate to produce MAs(III) antibiotic at low concentrations in *Nostoc*, and since there seems no ArsH in *Nostoc* (Chen et al., 2015a), NsArsI takes over at high concentrations to protect the cells from excessive MAs(III).

## Conclusion

We investigated the relationship between arsenic methylation and demethylation in cells that both *arsM* and *arsI* genes coexist. By assessing arsenic resistance and biotransformation by *E. coli* AW3110 expressing *NsarsM* or/and *NsarsI* and both genes’ regulation in *Nostoc*, we understand better the arsenic detoxification mechanisms in microorganisms.

## Author Contributions

JY, Y-GZ, and YY conceived and designed the project. YY, X-MX, and Y-QG did the experiments. YY and X-MX analyzed the data. YY, X-MX, Y-GZ and JY wrote the manuscript. All authors read and approved the final manuscript.

## Conflict of Interest Statement

The authors declare that the research was conducted in the absence of any commercial or financial relationships that could be construed as a potential conflict of interest.
